# The Roulette Metaphor in Living with Type 1 Diabetes: Semiotic Dynamics and the Regulation of Self-Experience in Christin’s Case

**DOI:** 10.1007/s12124-026-10016-1

**Published:** 2026-07-16

**Authors:** Juan José Cleves-Valencia

**Affiliations:** Department of Developmental Sciences, Cognition, and Neuroscience, Universidad del Valle, Santiago de Cali, Colombia

**Keywords:** Cultural psychology, Semiotic mediation, Type 1 diabetes mellitus, Affective semiosis, Meaning-making, Case study

## Abstract

Different theoretical developments in Cultural Psychology have advanced the understanding of the semiotic mediation of human experience, with particular emphasis on how cognition and affect are intertwined. Simultaneously, the application of Cultural Psychology to health contexts remains notably less developed, particularly in relation to the study of conditions involving multiple therapeutic demands within medical treatment. In this context, the present study aims to understand the configuration of the experience of medical treatment in Type 1 Diabetes Mellitus through the analysis of the organization, regulation, and interaction of affective semiosis and meaning-making processes. Methodologically, an idiographic perspective was adopted through the microgenetic analysis of in-depth interviews within a case study. The analysis is developed through the use of metaphors, understood as semiotic configurations that articulate the experience of the Self. A central metaphor, “the daily roulette”, and four peripheral metaphors serving structuring and operational functions were identified. Results show that experience is organized around an affective field of contingency that guides the interpretation of bodily variations. Processes of meaning-making are identified as operating through micro-stabilizations of meaning making orienting action in concrete situations. The study deepens the theoretical understanding of the articulation between the body, affective semiosis, and meaning-making, suggesting specific properties of signs regulation of experience. Within Cultural Psychology applied to the field of health, it also offers insights into how individuals regulate their experience of treatment in contexts of bodily uncertainty, thereby informing the development of approaches more attuned to patients’ affective dynamics.

## Introduction

Type 1 Diabetes Mellitus (T1DM) is a chronic and incurable health condition whose everyday management involves a multicomponent medical treatment that unfolds continuously within the changing flow of daily life activities and interpersonal relationships across diverse and shifting social contexts (ADA, 2023; IDF, 2021; Roncancio-Moreno et al., 2026). Living with T1DM requires attending simultaneously to metabolic variations, bodily sensations, therapeutic requirements (such as insulin administration, glucose monitoring, and regular medical check-ups) and to ordinary activities of daily life -such as studying, engaging in physical activity, working, eating, moving between places, or interacting with others- which continuously reshape the demands of treatment (Chatwin et al., 2021; Coffen, 2009).

In this context, managing the illness involves an ongoing reading of bodily sensations that are configured in relation to cultural artifacts and conventional signs. The perceived level of vital energy (level of activity, exhaustion, fatigue), tremors, sweating, or dizziness initially appear as sensible qualities of the body that require interpretation and already function as representamina in relation to possible metabolic states as objects. This interpretation does not occur directly, but rather through systems of semiotic mediation in which artifacts such as the glucometer, the syringe, or glucose-level logbooks also intervene, introducing new forms of stabilization and transformation in the representamen-object relation. Within these interpretive chains, signs refer to objects and generate interpretants oriented both toward action upon the treatment and toward the signification of the Self in its past, present, and future dimensions, within concrete contexts of everyday life. From this perspective, treatment can be understood as a continuous interpretive activity, configuring processes of anticipation and regulation of experience (Branco & Valsiner, 2010; Cleves-Valencia et al., 2025; Tateo, 2024). The semiotic mediation of the experience of illness, in addition to enabling the identification or anticipation of possible bodily crises, allows signals to be recognized, differentiated, and situated within temporal sequences in order to orient action (Zittoun, 2007, 2011; Zittoun, 2023). In this way, it contributes to sustaining a relatively stable relationship with one’s own body and with the surrounding world, which may become threatening, contingent, or unpredictable, with the potential to generate feelings of paralysis, helplessness, frustration, or, alternatively, an active search for transformation or transcendence (Cleves-Valencia & Ocampo-Cepeda, 2018; Frank, 1995; Kleinman, 2020; Sontag, 2005). From this standpoint, both illness and its treatment can be understood as psychological processes of meaning-making mediated by signs, offering a renewed perspective in contrast to approaches that position them in overly behavioral terms (Valsiner 2021a, 2024, 2021b; Salvatore, 2016).

In contrast, a large portion of the specialized literature has approached the management of T1DM from perspectives focused on treatment adherence, self-care, health behavior, or behavioral regulation, in which the primary aim largely consists of identifying psychological variables associated with clinical health outcomes (AlBurno et al., 2022; Álvarez et al., 2021; Campagna et al., 2020; Cervantes & Romero, 2023; Cleves-Valencia et al., 2024; Datye et al., 2015, 2017; International Hypoglycemia Study Group, 2015; Mansour et al., 2023; Prikken et al., 2019; Shahbazi et al., 2018). Although the fields of health and health psychology have produced a substantial body of empirical research, they often seem to convey an instrumental reading of human beliefs and behaviors. These tend to be understood in terms of means for achieving specific ends, namely health outcomes such as metabolic control, glycemic control, and the prevention of physiological complications (Ogden, 2018; Crossley, 2000). Similarly, core symptoms of the condition -such as hypoglycemia- are primarily treated as indicators of physiological states that require correction, whether for metabolic control or for quality of life (Floyd et al., 2016; Gunns & Leach, 2020; Helgeson, 2021; Lizarzaburu et al., 2020; Ly et al., 2014; Mahler et al., 2022; Miller et al., 2020; Nagl et al., 2022; Varni et al., 2018; Verbeeten et al., 2021). In contrast with the expansion of empirical-analytical approaches in health and applied health psychology, perspectives grounded in cultural-semiotic psychology and, in particular, idiographic studies focused on processes of sense-making, remain considerably less frequent. This asymmetry in the research corpus is not incidental, but reflects the epistemological, theoretical, and methodological complexity involved in studying human experience as a dynamic process of semiotic mediation, grounded in the depth of qualitative data derived from cases that enables the subsequent development of analytical generalizations (Salvatore & Valsiner, 2022).

More recent theoretical developments by pioneering authors working in cultural psychology and semiotics, such as De Picione, Salvatore, Valsiner, and Zittoun, have made it possible to understand how experience is organized through relations among signs that regulate the relationship between persons and the world (Valsiner 2007, 2014, 2021a). However, a particularly fertile field remains open for the study of the affective and embodied properties of signs and their role in the regulation of experience in contexts of uncertainty (Branco & Valsiner, 2010; Roncancio-Moreno et al., 2023). With varying theoretical nuances, these processes have been referred to as affective semiosis. In contrast to, yet intertwined with meaning-making, affective semiosis broadly refers to signs that become hypergeneralized, that operate continuously at a non-conscious level, and that have sufficient force to orient the development of the Self (De Picione & Tossici, 2025; Salvatore et al., 2025; Salvatore & De Picione, 2023; Valsiner, 2024, 2021b; Zittoun, 2023). At the same time, empirical contributions within cultural-semiotic psychology addressing a specific condition such as T1DM remain, to our knowledge, very limited. This is mainly due to four reasons. First, T1DM has a markedly lower prevalence than Type 2 Diabetes Mellitus: while the former is typically autoimmune in nature, the latter is associated with personal, familial, and sociocultural lifestyles prevalent in contemporary forms of life (IDF, 2021). Second, this reflects the theoretical specificity of a field that has traditionally been more concerned with the development of the Self under conditions of health. Third, the field of health has historically been the domain of clinical or biomedical research. Finally, it appears that insufficient attention has been given to what a condition such as T1DM can reveal about the properties of signs in the regulation of human experience. That is, what can be learned, through this chronic condition, about the body- self- world relation and about the regulatory properties of signs in both conscious and non-conscious life.

In the specific case of T1DM, some studies have suggested how affective semiosis operates in crises generated by hypoglycemia and the implications it may have beyond the immediate sensation of bodily discomfort (Cleves-Valencia et al., 2025). In this context, hypoglycemia appears as a hypergeneralized affective field, with considerable force in channeling the development of the Self at levels that are difficult to communicate verbally, insofar as they do not function as discrete signs (as emotions do, for example, which can be identified and named). Another very recent study, situated at the intersection of dialogical and cultural perspectives, suggests that a central therapeutic requirement for managing the condition, such as dietary regulation, entails a shift from a shared experience at the family level toward the intimate bodily dialogue of the person living with the diagnosis, within a dialogical movement involving continuity, change, and familial conflict (Roncancio-Moreno et al., 2026). In this way, within the field of cultural psychology applied to the study of a condition such as T1DM, there exists an initial set of contributions addressing specific dimensions of the illness -namely, hypoglycemia and dietary management-. To our knowledge, however, there is no contribution that accounts for the relation between the semiotic regulation and organization of the overall experience of this condition while simultaneously offering a novel contribution to the understanding of the life of signs (i.e., their regulatory dynamics). In this regard, it is important to suggest that asking how signs organize experience or anticipate what occurs in the body shifts the focus from a primarily clinical domain (as it is often framed in the health field) toward a broader perspective that situates it as a challenge for psychological approaches concerned with the continuity of the Self and its semiotic regulation; that is, for what properly belongs to the field of cultural psychology (Valsiner 2014, 2021a, 2021b, 2024). From this perspective, further understanding how processes of affective semiosis and meaning-making are organized, regulated, and interrelated becomes central both for the psychological study of chronic conditions and for the advancement of the field itself.

Given the empirical and theoretical gap in the field of cultural psychology, the present study seeks to contribute to the dialogue between the empirical and the theoretical by showing how the experience of the everyday management of medical treatment in T1DM can be rendered intelligible through an idiographic analysis of a case in which the roulette metaphor emerges from the participant’s own voice. This metaphor makes it possible to observe how the experience of treatment may be organized around uncertainty, discontinuity, and partial loss of anticipation, thereby constituting a vantage point for examining the regulation of experience through semiotic processes, as well as the regulatory functions of signs in contexts of chronic illness. Accordingly, the present study is guided by the following question: *What does the case of Christin*,* and the roulette metaphor*,* make it possible to understand about the semiotic mediation of the experience of medical treatment in T1DM?* To address this question, the present work aims to *understand the configuration of the experience of medical treatment in the chronic condition of T1DM through the analysis of the organization*,* regulation*,* and interaction of affective semiosis and meaning-making processes*,* with the purpose of offering an empirical contribution to Cultural Psychology*,* with theoretical implications for its application in the field of health.*

## Methodology

The present study is grounded in an idiographic approach. From this perspective, psychological phenomena are understood as configurations that emerge in the interrelation between the subject, the world, and the systems of signs that mediate this relationship (De Picione et al., 2019; Salvatore & Valsiner, 2022; Valsiner, 2017). In this sense, the idiographic approach does not aim to describe directly identifiable contents, but rather to analytically abstract the semiotic organization of experience. The design is based on a single retrospective case (Zittoun et al., 2022). Individual cases are not understood as particular instances of previously defined general categories, but as privileged contexts for identifying general processes in the particular (De Picione, 2015). The analytical approach is guided by a microgenetic perspective oriented toward understanding how experience is organized, reconstructed, and regulated through signs in its present actualization (Valsiner, 2014). Although the case is retrospective, the analysis focuses on the moment-to-moment configuration of meaning-making processes as they emerge within the participant’s accounts, rather than on the chronological reconstruction of past events themselves. The analysis is organized around the identification of a central metaphor and a set of peripheral metaphors, analytically categorized as serving structuring and operational functions, which make it possible to understand the regulation of the Self in contexts of variability and its relation to the medical treatment of T1DM (Guenther, 2023).

### Participant

The participant, referred to as Christin (pseudonym), is a 27-year-old woman diagnosed with T1DM at the age of 14 (for further information about her life context, see the Results and Analysis section).

### Data Construction

Data were constructed through seven in-depth interviews guided by a flexible protocol, in order to facilitate the participant’s verbal expression within her own frameworks of meaning (Packer, 2018). This protocol were reviewed by an expert researcher and psychotherapist with more than 35 years of experience. It was organized around a set of thematic axes intended to facilitate the exploration of the participant’s experience in relation to T1DM (See Table 1). In accordance with the principles of non-directiveness and recursive qualitative inquiry, the relevance and depth of each theme varied throughout the interview process depending on the participant’s accounts.

**Table 1:** Thematic organization of the interview protocol. Source: Author's own elaboration

Thematic organization of the interview protocol	
Thematic axis	Exploratory dimensions
*Family and relational contexts*	Family relationships with significant others, forms of support and conflict in everyday interactions, and the relational processes through which meanings and orientations toward living with T1DM are mediated.
*Bodily experience and health–illness perception*	Bodily sensations, experiences of well-being and discomfort, perceptions of bodily changes, meanings attributed to symptoms, and subjective understandings of health and illness.
*Developmental transitions and life trajectory*	Significant experiences across childhood, adolescence and adulthood; educational and occupational transitions; changes and transformations associated with living with T1DM over time.
*Critical events and disruptive experiences*	Hospitalizations, metabolic crises, emotionally significant events, situations of uncertainty, loss, fear, or disruption, and their implications for the Self experience.
*Everyday regulation of treatment and anticipation of bodily changes*	Processes through which treatment is organized in daily life, including insulin administration, glucose monitoring, food-related practices, physical activity, anticipation of possible bodily states, decision-making, and strategies for managing uncertainty.
*Social participation and future orientations*	Occupational activities, friendships, intimate relationships, personal projects, expectations and imagined futures in relation to living with T1DM.

Data production was conceived as a dynamic and recursive process, and the exploration of the object of study was progressively refined (Zittoun, 2011). Interviews were recorded using the Easy Voice Recorder application and fully transcribed using OTranscribe software. The unit of recording corresponded to participants’ verbalizations. Additionally, field notes were produced, documenting questions, comments, and preliminary analyses by the researcher. The interview process continued until saturation was reached, understood as the sufficiency of information in relation to the research objective (Willig, 2012). This criterion was dialogically established with the support of the expert researcher and psychotherapist (Cornish et al., 2014).

### Data Analysis Procedure

The analysis was oriented toward understanding the configuration of the experience of treatment in T1DM through the organization, regulation, and interaction between processes of affective semiosis and meaning-making (Branco & Valsiner, 2010). In line with this objective, the analytical procedure was developed through several interrelated phases:

#### **Phase 1. Immersion and delimitation of the analytical field**

A thorough reading of the transcripts was conducted, identifying relevant units of sense based on their potential to account for the experience of treatment, with the support of Atlas.ti software. This phase enabled an initial approximation to the processes of semiotic mediation present in the material.

#### **Phase 2. Development of graphical representations**

**Fig. 1:**
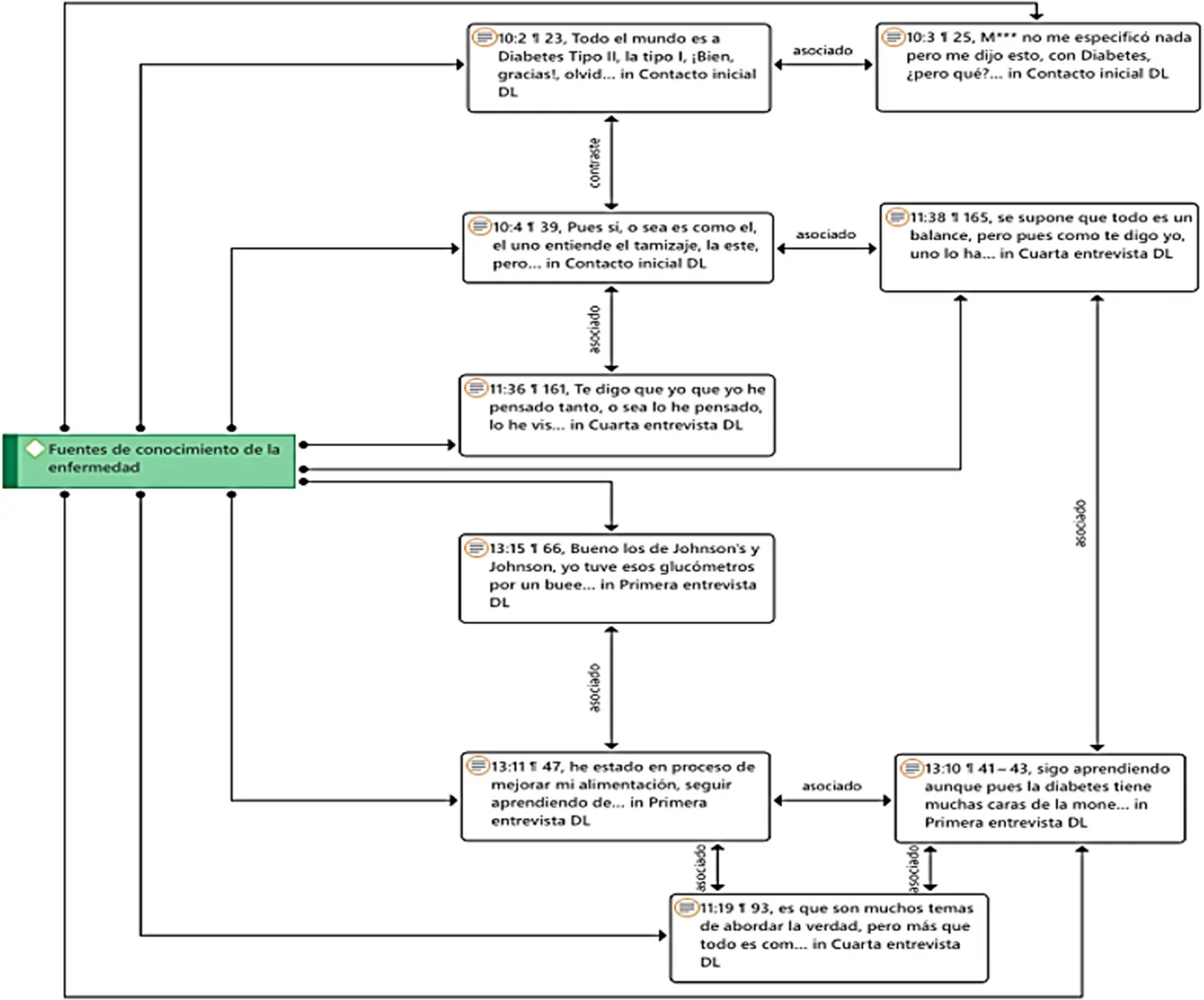
Modes of understanding the illness. Source: Author’s own elaboration

Networks of associations based on the verbatim were constructed to organize the information and to provide a preliminary visualization of relations among signs (see Fig. 1). These representations were conceived as tools for case construction and for the analysis carried out in Phases 3 and 4.

#### **Phase 3. Identification of semiotic configurations**

Through iterative readings, chains of signs within the selected excerpts were explored, attending to their functioning as representamina that refer to objects (bodily states, treatment-related events, everyday situations) and generate interpretants oriented toward action, anticipation, or the regulation of experience (Tateo, 2024).

#### **Phase 4. Analysis of metaphors as semiotic configurations**

In correspondence with the previous phase, the analysis privileged metaphors, understood as affectively charged semiotic configurations through which individuals organize, interpret, and regulate lived experience under conditions of uncertainty (Branco & Valsiner, 2010). This perspective is grounded in the proposition that “the central nature of human communication through signs is its flexibility and openness both at a given level of abstraction and between different levels” (Valsiner, 2014, p. 57). Accordingly, metaphors are not approached merely as rhetorical devices, isolated figurative expressions figurative or cognitive structures (Bourke, 2014; Lakoff & Johnson, 2003; Sontag, 2005), but as semiotic processes that participate in the organization of experience. As proposed by Valsiner, “different forms of language use -proverbs, similes, metaphors- operate to enhance the process of abstraction” (Valsiner, 2014, p. 57), allowing persons to establish relations between bodily sensations, previous experiences, present situations, and anticipated futures. From this standpoint, metaphorical processes participate in meaning construction, which “operates in the direction of generalization” (Valsiner, 2014, p. 62), giving rise to hypergeneralized sign fields that may “capture the whole person” (Valsiner, 2014, p. 57). Thus, metaphors contribute to the semiotic regulation of self-experience by organizing affective orientations, interpretive possibilities, and forms of action across irreversible time (Valsiner, 2014, p. 126). In this sense, “the person repositions oneself by relating a present experience with one from the past, and the past is brought into the present”, thereby establishing continuities that support orientation under conditions of uncertainty (Valsiner, 2014, p. 58).

Within this framework, the body occupies a central position because “the human body is the arena for the dual process of internalization and externalization” (Valsiner, 2014, p. 63). Consequently, metaphorical processes emerge through embodied engagements with the world mediated by signs and culturally shared symbolic resources. This perspective makes it possible to account for the unity of cognition and affect, as well as for the inseparability of body, mind, and culture in the regulation of experience (Roncancio-Moreno et al., 2023). Accordingly, metaphors were conceived as semiotic configurations apprehensible in language, action, and broader patterns of experience (Roncancio-Moreno et al., 2025). Some were identifiable through explicit figurative expressions, whereas others emerged through temporally distributed chains of signs involving bodily interpretation, artifact-mediated processes, and the reconstruction of experience with significant others (Zittoun, 2023). Metaphors were selected based on three criteria: (a) their affective potential, (b) their capacity to articulate multiple dimensions of experience, and (c) their potential to make visible processes of semiotic regulation. Based on these criteria, one central metaphor, “the daily roulette,” and four peripheral metaphors, analytically categorized as serving structuring and operational functions, were identified.

### Ethical Considerations

The study was conducted in accordance with the ethical principles governing the practice of psychology in Colombia (Law 1090 of 2006), ensuring conditions of confidentiality, privacy, and participant well-being. Pseudonyms were used, and identifying information was removed. Participants were informed about the objectives, procedures, and scope of the study, and voluntarily provided informed consent. Their autonomy to continue or withdraw from the study at any time was also respected. As part of the process, a feedback session was conducted in which general aspects of the analysis were shared with participants, fostering an ethical relationship grounded in recognition and reciprocity.

## Results and Analysis

After presenting the general context of Christin´s case, the analysis is organized around the central metaphor, which is introduced first, followed by its four interrelated peripheral metaphors. Each metaphor is presented in a separate section and analyzed semiotically in articulation with excerpts from the verbatim. The metaphor of the daily roulette occupies a central position because it condenses the overall experience of living with T1DM and organizes multiple bodily, affective, temporal, and action-related dimensions. The remaining metaphors are analyzed in relation to the central metaphor as part of an interconnected system in which structuring metaphors make visible relatively stable organizing principles through which experience is interpreted and regulated, whereas operational metaphors reveal semiotic operations through which bodily events are interpreted, uncertainty is managed, and action is oriented in everyday life. Rather than functioning as a totalizing description, the roulette metaphor condenses recurrent experiences of uncertainty, unpredictability, and limited anticipation. At the same time, other dimensions of experience -including efforts to construct stability, verify bodily states, or restore temporal continuity- are made visible through additional metaphors that complement and specify aspects of experience not captured in their entirety by the roulette metaphor alone (Table 2). The table below presents the identified and analyzed metaphors, the corresponding verbatim excerpts with which they are articulated, and the inclusion criteria that guided their integration into the case analysis (Table 1).

**Table 2:** System of metaphors in the regulation of self-experience. Source: Author’s own elaboration

System of metaphors in the regulation of self-experience and the medical treatment of T1DM			
Type of metaphor	Identified metaphor	Associated excerpt	Rationale for selection
*Central Metaphor*	The daily roulette	17:41 ¶ 94	It condenses the overall experience of treatment and articulates multiple temporal, affective, bodily, and action-related dimensions.
*Structuring Metaphors*	The brick and the foundations	11:39 ¶ 165	Due to the specific aspects it reveals about meaning-making and affective semiosis and its relation to the search for stability in the Self.
	The disturbance of the mind	16:4 ¶ 40–43	Because of its role in the effort to preserve homeostasis in the face of the emergence of thoughts and affects.
*Operational Metaphors*	The verification of experience (“Am I seeing this correctly?”)	15:24 ¶ 173	It highlights the relational and artifact-mediated processes required for interpreting bodily signs during moments of symptomatic crisis.
	The loss of the “when” (“At what time?”)	17:9 ¶ 89–92	Given its capacity to reveal temporal discontinuity in experience and its implications for the anticipation and regulation of action.

### General Context

Christin (pseudonym) is a 27-year-old woman diagnosed with T1DM at the age of 14. She lives within a family composed of her parents and younger brother. Her father works in support services at a public university, while her mother has been a homemaker since the birth of her second child. Christin completed undergraduate and postgraduate studies in economics and had recently started a new professional position at the time of the interviews. Prior to this, her participation in the labor market had been intermittent and mainly informal. She reported no marital or cohabiting relationships and described a social life centered primarily on family members, while maintaining contact with a small number of acquaintances through social media and digital communication technologies. Shortly after birth, she underwent pancreatic surgery due to nesidioblastosis and subsequently participated in different medical and therapeutic processes. She received the diagnosis of T1DM during adolescence and reported at least one hospitalization associated with a hyperglycemic episode. Christin described growing up largely in the company of adults and attending schools with relatively small numbers of students during childhood. She also reported experiencing a depressive episode following the death of her maternal grandmother. At the time of participation, she expressed an interest in numerology, calendar systems, and spiritual forms of knowledge.

### Results and Analysis by Metaphors

1. The daily roulette.

Excerpt 17:41 ¶ 94

*Well*,* lately it has been going up a lot; today… in a little while I’ll check to see how this whole roulette situation turns out -as I call it-*,* the daily roulette. I don’t like to call it anything else*,* the roulette. And*,* as I tell you*,* this illness is each day building*,* destroying what one… (inaudible)*.

*“… I used to think*,* almost*,* five years—almost five*,* four years—of being on this plan*,* of being on this insulin regimen*,* of being on this plan of waiting to see where the roulette wheel is going to land. And I tell her*,* you know what I think now? That diabetes is a daily struggle. I see diabetes as a daily battle; the day-to-day*,* the day-to-day.”*

The metaphor of the “daily roulette” introduces a mode of organizing experience in which the becoming of the body and of the treatment is configured under the quality of chance. The expression “to see how this whole roulette situation turns out” situates experience within an open horizon in which the outcome does not appear as anticipated, but rather unfolds as an indeterminate possibility. At this point, the roulette functions as a sign whose object does not refer solely to glycemic variability, but to the way in which such variability is lived: as contingency emerging in the course of the day. This interpretation is reinforced by other moments in Christin’s accounts in which treatment is described as “waiting to see where the roulette wheel is going to land” and as a “daily battle”, suggesting that uncertainty is not experienced as an isolated event but as a recurrent condition organizing everyday engagement with T1DM. However, the roulette metaphor provides a way of unifying heterogeneous experiences under a single affective quality. Here, affective semiosis appears as an always-operating process that configures the background from which events are interpreted. The insistence in “I don’t like to call it anything else” introduces a moment of stabilization in the semiotic chain and expresses Christin’s agency. The metaphor does not appear as a circumstantial denomination, but as a form that organizes experience. In this sense, the interpretant configured through the “roulette” orients the relation to the treatment and to one’s own body under a logic of uncertainty, in which anticipation is articulated in a particular way: not as a projection of relatively differentiated scenarios, but as an openness to a broad spectrum of possible outcomes.

The sequence “each day building, destroying”, in turn, introduces a polar dynamic. These poles do not appear as articulated moments, but as qualities that coexist in the experience of everyday life. Construction and destruction operate here as signs that condense states of the relation to the treatment, referring simultaneously to actions carried out, bodily effects, and self-related meanings. Within this chain, each of these signs can become a representamen for new interpretations, extending semiosis in different directions. Affective semiosis becomes evident by conferring qualitative unity upon experience. The “roulette” introduces an affective tone through which events acquire sense, as a field encompassing the daily experience as a whole. From there, meaning-making unfolds in relation to that field, producing interpretations organized around variability, contingency, and openness of outcomes. Anticipation, in this context, does not disappear, but takes on a particular configuration: it is oriented less toward the differentiation of specific signs and more toward a stance in relation to a range of possibilities. In this way, the experience of treatment is organized within a temporality in which the present is permeated by the expectation of continuous variation, and in which signs do not stabilize solely as indicators of differentiated states.

2. The brick and the foundations.

Excerpt 11:39 ¶ 165

*Many times*,* just because one knows certain things doesn’t mean one knows everything*,* no one knows everything about a specific topic*,* no one. Anyone who says they know everything about everything*,* or that they know a topic 100%*,* is a real liar. Because you may know one concept*,* but if you don’t know the next one*,* then what? Doesn’t the whole brick structure you so carefully organized collapse*,* doesn’t it fall apart? Why? Of course it does. But if there are no solid foundations*,* what can you do? Nothing. If the concept is there but it’s unstable*,* then what? Doesn’t what one has built*,* in one way or another*,* start to fall apart? And if you… put me*,* like*,* within a grid*,* at least for me*,* but in some things I’ve reflected and I say*,* well*,* there are grids one has to follow*,* but not all of them.*

*“… I mean*,* diet combined with exercise*,* diet… I mean*,* looking at every side of the table and being able to organize each one*,* but I haven’t taken the time*,* I haven’t done it*,* I haven’t been disciplined about it. It’s left me*,* as they say*,* in a kind of limbo of*,* of things… I mean*,* of thoughts*,* of feelings*,* of*,* of emotional stability*,* and of bringing them all together into one piece and saying*,* well*,* this is the first brick I lay today*,* this is the second*,* this is the third*,* building step by step*,* but with solidified information.”*

In this excerpt, the experience of treatment and the knowledge associated with the condition are organized through a metaphor that refers to a process of construction grounded in foundations and built through bricks, involving effort. Through this figuration, knowledge about T1DM appears as a structure that is progressively built, in which each element depends on the solidity of the previous ones. The expression “the whole brick structure you so carefully organized collapses” introduces a relation of interdependence among the signs that compose this knowledge, in which the absence or instability of one of them has effects on the whole. In semiotic terms, this metaphor makes it possible to observe how the operation of affect and thought in Christin is oriented toward the search for articulation among signs. The structuring metaphor reappears elsewhere when Christin refers to “the first brick”, “the second”, and “the third”, emphasizing the gradual and cumulative character of the semiosis required to achieve the stability of the Self in relation to treatment, emotions, and knowledge.

Within this process, oscillations between the concrete and the figurative are notable, that is, between “bricks” and “concepts.” This displacement constitutes an operation that allows the participant to articulate different levels of the experience of treatment. “Bricks” refer to a dimension of materiality and assemblage, in which knowledge appears as something built piece by piece, while “concepts” introduce a level of abstraction that seeks to organize and give coherence to those pieces. In this dynamic, meaning-making is not limited to the accumulation of information, but involves the need to establish relations among signs of heterogeneous nature and different properties that configure experience. The movement from “bricks” to “concepts” thus signals an attempt to integrate lived experience -sensations, practices, medical instructions- into more general structures that may orient action. At the same time, the metaphor introduces the ongoing possibility of instability. The expression “it’s unstable” does not refer to a definitive collapse, but to a dynamic quality in which the experience of the Self can still be sustained. At this point, affective semiosis becomes present as the field that confers upon this construction a tone of precariousness or vulnerability, from which processes of meaning-making unfold. The reference to the “grid” introduces an additional element in the organization of experience. It appears as a form of structuring and containment that could provide stability, yet its adoption is not total: “there are grids one has to follow, but not all of them”. At this point, meaning-making is oriented toward a selective relation with available frameworks of organization; it is expressed as a positioning in relation to a metaphorical content and suggests an expression of agency. This points to a dynamic in which the regulation of experience does not depend solely on the incorporation of external structures, but on the selection of the conditions under which experience is regulated. In relation to the central metaphor of the roulette, the “brick” and the “foundations” make it possible to observe a complementary movement in the configuration of experience. Whereas the roulette organizes the field under the quality of contingency and variability, this metaphor introduces the search for forms of stabilization that allow for sustaining a certain continuity in interpretation and action.

3. The disturbance of the mind.

Excerpt 16:4 ¶ 40–43

*Interviewer: But when you feel exhausted, is that when your blood sugar is high, ordo you usually feel like that? Because you mentioned that at some point you neglected your health a bit, and I’m clear about that, but beyond that specific situation, have you experienced any kind of bodily change in terms of fatigue, or sleep, or any other manifestation that you notice? Christin: No, well, in periods, it’s not very constant, not very constant. Interviewer: And in the periods when it happens, why does it happen? Christin: Because I’ve gone past the indicated time, or because I do sleep but I feel stressed by thoughts, although now I’ve started to, like, think things through a bit and learn to manage certain psychological aspects so that my mind doesn’t get disturbed.*

*At least I try to balance my career with my spiritual interests because I’ve realized -and people often fall into that damn mistake- that sometimes you start thinking about everything in numerical terms. You end up thinking about everything quantitatively*,* even though there is a qualitative side that we often take for granted. And many times it is the qualitative side that changes*,* while the quantitative side does not*,* so it ends up feeling like a bottom with no way out.*

In this excerpt, the experience of the body and of treatment is organized through a temporality that appears as discontinuous: “in periods”. This way of naming introduces a broad segmentation of experience, in which bodily changes such as fatigue or sleep are grouped into states without precise internal differentiation. Within this framework, the metaphor of the disturbance of the mind introduces an organizing axis of experience in which the psychological dimension is figured as a space susceptible to alteration. The interpretant that is configured orients action toward the management of “thoughts” and of “certain psychological aspects”, in a direction that privileges the preservation of a certain stability. This search for the stability of the Self also appears when Christin describes the tendency to think about everything “in numerical terms” and to privilege quantitative over qualitative dimensions of experience. Such an orientation suggests an effort to stabilize experience through forms of calculation, categorization and control. Yet, when these forms prove insufficient, experience may acquire the quality of “a bottom with no way out”, a metaphor conveying a loss of orientation, the feeling of being trapped within one’s own interpretive field, and a diminished sense of agency.

Within the semiotic chain that unfolds, thoughts, stress, and fatigue are linked without an explicit differentiation between their conditions of emergence. Rather, these elements appear as components of a single experiential field. At this point, affective semiosis becomes present as the quality that unifies these elements under a common tone, in which stress does not appear merely as a cognitive content, but as a form that traverses the experience of both the body and thought. Meaning-making, in turn, is oriented toward the regulation of this field through reference to work on oneself: “to think things through a bit” and “to manage certain psychological aspects”. These signs introduce a direction in the organization of experience that seeks to establish some form of mediation and distancing in relation to variability. Such mediation unfolds as a general orientation toward the maintenance of stability. In relation to the central metaphor of the roulette, this excerpt makes it possible to observe how experience is not organized solely around the unpredictability of outcomes, but also around the need to sustain a balance in the Self.

4. Am I seeing this correctly? The verification of experience.

Excerpt 15:24 ¶ 173

*Look*,* I’m going to tell you a story. It turns out that once I started exercising -not just exercising*,* but intense exercise*,* intense- and at one point I felt something*,* I felt something*,* but I thought it was just something momentary*,* you know? Just something passing. I was with a relative*,* a cousin -he’s younger than me- and I said to him*,* “Look*,* look*,* what’s my blood sugar reading?” And it showed 38. And I said*,* “What am I seeing? Am I seeing this wrong? Come here*,* run*,* check this*,*” I told my cousin. He said*,* “Yes*,* yes D*,* you’re seeing it right.” And I told him*,* “Bring me this*,* and don’t tell anyone*,*” but obviously*,* because of his age… my grandmother*,* I didn’t want to worry her.*

In this excerpt, the expression “I felt something” introduces a sensible quality that remains initially indeterminate and is first interpreted as “something momentary”. This initial interpretation does not stabilize the experience, but rather maintains it in a state of ambiguity, in which the relation between bodily sensation and meaning remains open. The glucose measurement introduces a new element into the semiotic chain by providing a conventional sign that refers to a specific bodily state. However, rather than closing interpretation, the result “38” produces a rupture in the continuity of experience, expressed in the question “What am I seeing? Am I seeing this wrong?” At this point, the sign itself becomes an object of questioning. Experience is not organized solely around what occurs in the body, but around the very possibility of trusting the signs that represent it. In this context, verification emerges as a central operation. The request for corroboration that Christin addresses to her cousin introduces a possibility that redistributes the interpretive load. The other does not appear merely as a companion, but as an instance of validation of experience. The cousin’s confirmation (“you’re seeing it right”) contributes to momentarily stabilizing the relation between representamen and object, enabling the reorientation of Christin’s action in response to hypoglycemia. This process highlights that meaning-making is not sustained solely through the individual’s relation to signs, but is configured within relational networks in which interpretation can be shared, contrasted, and confirmed. Moreover, in Christin’s instruction to her cousin, “don’t tell anyone”, it becomes evident that experience is not only verified, but also managed in terms of to whom it is made visible and under what conditions. At this point, the regulation of experience involves not only the stabilization of meaning, but also the delimitation of its circulation within the social field. In relation to the central metaphor of the roulette, this excerpt shows how unpredictability is not limited to variations in bodily states, but also extends to reliance on signs, on others, and on the artifacts that mediate the experience of the body and medical treatment, such as the glucose meter.

5. “At what time?”: The experience of the loss of the “when”.

Excerpt 17:9 ¶ 89–92

*Interviewer: And for some people it can be frustrating that they follow the diet and still their blood sugar drops or rises*,* how has that been in your case?*

*Christin: Well*,* when mine has dropped from exercising -once it went down to 38*,* but it was while exercising- I don’t know*,* I mean*,* I felt out of breath*,* but I didn’t really think much of it. When I checked my blood sugar*,* that’s when I said*,* “But why? At what time did this drop happen?” I stayed still because it was below 50*,* so I stopped*,* I mean*,* I finished the routine and then I checked it. When I checked it*,* I told my cousin*,* I said*,* “Look at this result*,*” and he said*,* “No*,* look at this*,*” and I said*,* “This! And at what time?!”*

*Interviewer: What do you think about that, and how do you feel about it? Christin: Well, honestly, I feel—I mean, when I think about it, I say, okay, it’s excessive exercise, or, well, I should do intense exercise but not for so long.*

In this excerpt, the experience of treatment is organized around a rupture in temporal anticipation condensed in the question “And at what time?” This suggests a discontinuity in the possibility of situating bodily events within a temporally comprehensible sequence in experience. The narrated sequence introduces several elements that make this configuration observable. On the one hand, intense physical activity appears as a possible antecedent; on the other, the initial bodily sensation (“I felt out of breath”) is not immediately interpreted as a sign of hypoglycemia. Only retrospectively, through measurement, does the event acquire a defined status, and the interpretive operation makes it possible to know “what” is happening, but not “when” or “how” it came to be configured.

At this point, meaning-making is oriented toward the retrospective reconstruction of experience. The question “At what time?” signals an attempt to reinscribe the event within a temporal sequence, and a gap remains between the occurrence of the phenomenon and its integration into a meaningful sequence. The exclamation “This! And at what time?!” introduces a quality of bewilderment that configures the very way in which the event is lived and orients the need to find an explanation that may restore some degree of continuity for the Self. Toward the end of the excerpt, an attempt to reorganize experience emerges. At this point, meaning-making is reconfigured around the formulation of specific causal links that allow Christin to anchor the experience in an explanation sufficient to orient action. Rather than an reconstruction of what occurred, the meaning produced introduces a localized reorientation of future action, in which interpretation operates as a resource for restoring the capacity to act in the face of experience. The loss of the “when” marks a point at which meaning-making unfolds retrospectively, seeking to reestablish connections that were not available at the moment of action. In this way, the experience of treatment is configured in a tension between the occurrence of events that cannot be anticipated and subsequent attempts to integrate them. 

## Discussion and Conclusions

Analyzing medical treatment experience in contexts of chronic illness offers a privileged field for examining the semiotic mediation of the relationship between the body, the world, and the Self. Based on Christin’s case, it is possible to contribute to the understanding of how processes of experience regulation are configured under conditions of bodily variability, particularly in the articulation between affective semiosis and meaning-making. This case also makes it possible to situate a broader theoretical discussion on the ways in which the subject sustains the continuity of experience in contexts of uncertainty. At a theoretical level, the field has increasingly approached affect as an immediate and ubiquitous process, clearly differentiated from the reflective construction of meaning (Tateo, 2024; Valsiner, 2024; Zittoun, 2023). This interest has given rise to three main models, which are discussed below.

First, the semiotic regulation model proposed by Branco and Valsiner introduces the concept of affective hypergeneralization to explain how affectively charged signs, at different levels of generalization, organize experience and participate in the development of the Self (Branco & Valsiner, 2010). This hierarchical system includes less differentiated levels of regulation, linked to physiological sensations, as well as more abstract levels that can guide action, within a continuum of regulation and semiotic mediation (Branco & Valsiner, 2010; Valsiner, 2014; Valsiner, 2024). These developments make it possible to interpret the central metaphor of the roulette in Christin’s case as a semiotic configuration that acquires hypergeneralized characteristics, organizing the field of experience through a dominant affective quality -in this case, contingency- which orients the interpretation of multiple situations related to the Self and medical treatment.

Second, Zittoun proposes a three-dimensional model of mental activity that integrates conscious and non-conscious dynamics, semiotic mediation, and temporality (Zittoun, 2023). Her work highlights how even non-conscious processes can be mediated by complex signs. The author’s theoretical developments suggest that meaning-making is not limited to the reflective elaboration of experience, but rather constitutes an ongoing process through which individuals articulate heterogeneous elements -perceptions, memories, affects, expectations, and cultural artifacts- in order to sustain a certain experiential consistency (Zittoun et al., 2013). From this perspective, meaning-making involves producing differentiations that allow experience to be discretized and rendered communicable, anticipable, and relatively stable (Zittoun, 2006, 2007). Within the author’s theoretical reasoning, the relation between rupture and transition takes on a particular form. While Zittoun has shown how ruptures may give rise to processes of transition through the reorganization of meanings, the case analyzed here makes it possible to propose the existence of configurations in which experience is organized under conditions of recurrent rupture, without necessarily leading to stabilized transitions. Instead, localized processes of differentiation are observed, which can be understood as micro-stabilizations of meaning: partial configurations that allow individuals to continue acting in the immediate situation without necessarily consolidating into generalized interpretive frameworks. These micro-stabilizations are not failures of the meaning-making process, but specific forms of its functioning in contexts where experience demands constant reorganization. They operate as a regulatory process in which broad affective fields and localized processes of meaning-making are dynamically intertwined. This form of organization does not imply an absence of meaning, but rather a specific configuration in which the continuity of experience is sustained under conditions of recurrent uncertainty. The analysis of Christin’s case suggests that, in contexts of persistent bodily variability, the articulation of heterogeneous aspects of experience does not always lead to the stabilization of broad, generalized configurations of meaning.

Third, the work on affective semiosis and perceptual semiosis, in their different variations, as proposed by Salvatore and De Picione, is particularly useful for analyzing how the experience of the Self and medical treatment is configured in a case such as Christin’s (Salvatore et al., 2025; Salvatore & De Picione, 2023; Salvatore, 2016). Affective semiosis, by operating through global tonalities of experience, organizes the field in a rapid, diffuse, and wide-ranging manner, without requiring precise internal differentiations. In turn, perceptual semiosis introduces distinctions that allow elements of experience to be discriminated and action to be oriented (De Picione & Tossici, 2025). The results of the case support these developments by indicating that they do not appear as a balance between both modes of functioning, but rather as a complex interaction in which differentiation emerges partially within affective fields that maintain their primacy. In this way, the case suggests that processes of anticipation regarding medical treatment and of imagination, understood as an expansion of reality, are not configured solely as differentiated projections of the future, but as an affective disposition toward variability that allows action to be sustained when relations among signs do not fully stabilize (Zittoun et al., 2013).

At the empirical level, several studies have begun to explore meaning-making and, to a lesser extent, the affective dimension of experience in health contexts other than T1DM. However, given their objectives and theoretical orientations, these investigations do not articulate in an integrated manner the body, affect, and meaning-making. For example, a pioneering study on bariatric surgery highlights the role of social rejection and cultural prejudice in the configuration of the Self, but does not examine how embodied and affective experience functions as a medium of semiosis (Branco & Silva, 2019). More recent research has emphasized the role of metaphor and creativity in relation to bodily transformation, as observed in studies on heart transplantation and cancer prevention in young women, yet without addressing affective semiosis or its articulation with meaning-making (Pinheiro et al., 2025; Lemmo et al., 2022).

Within this state of knowledge, a recent study has opened a line of inquiry specifically focused on Type 1 Diabetes Mellitus. Through the analysis of a case, this work examined how an initial bodily sensation such as hypoglycemia may expand and hypergeneralize toward central aspects of identity (Cleves-Valencia et al., 2025). In that study, experience was organized around processes of progressive differentiation, in which signs derived from the symptom were subjected to operations of discretization, reorganization, and more deliberate articulation, enabling, on the one hand, the integration of experience into more stable configurations of meaning, and, on the other, the communication of such experience to other individuals. In contrast, the present case, rather than evidencing a predominance of differentiation processes, highlights the organizing weight of affective semiosis in the configuration of experience, expressed in terms of contingency and chance (condensed in the metaphor of the “daily roulette”). In this sense, the comparison between both cases allows for a more precise argument: the regulation of the Self in contexts of chronic illness does not follow a single logic of semiotic organization. Rather, it is configured through heterogeneous dynamics in which affective semiosis is always constitutive, although its mode of operation and degree of predominance vary. This variability, in turn, appears to point to qualitatively distinct forms of Self-regulation in the face of bodily uncertainty.

Although meaning-making entails the possibility of articulating experience into temporal sequences that allow for establishing relations between past, present, and future, the present case shows that such organization does not take the form of a continuous and differentiated sequence (Zittoun et al., 2013). On the contrary, the “when” of experience appears diffuse when it is reconstructed retrospectively. Christin´s case suggests an experience of the Self organized around forms of temporality in which the present acquires predominant weight, whereas the past and the future do not consistently operate as differentiated references. This temporal reorganization is consistent with the primacy of broad affective fields in the configuration of experience, insofar as temporal orientation relies more on the global quality of the experiential field, giving rise to forms of anticipation that are more open, less specified, and closely tied to the immediacy of experience.

Taken together, the findings have relevant implications for the field of cultural psychology and its dialogues with, and interventions in, the health domain. Under conditions in which patients must make constant decisions in contexts of bodily variability, the difficulty does not lie solely in a lack of information or in errors of interpretation, but in the complexity of the semiotic and temporal articulation of experience and in what this entails for understanding the body, the Self within the experienced health condition, and medical treatment. These findings point to the need to move toward the construction of a more robust case base, followed by their continuous comparison, in order to identify regularities in the semiotic organization of experience in contexts of chronic illness. Such steps are key for the progressive elaboration of theoretical principles concerning the regulation of the Self and the properties of signs under conditions of bodily uncertainty, which may, in turn, inform the development of principles for sociocultural psychological intervention in the field of health. In this sense, the challenge lies in articulating the recognition of the singularity of each experience with the need to generate frameworks of understanding that can guide decision-making in care settings.

## Data Availability

Data can be requested directly from the author.
